# Cross-Modality Diagnostic Agreement in Canine Medial Coronoid Disease in a Screening Population: Radiographs vs. CT in 424 Elbow Joints

**DOI:** 10.3390/vetsci12090883

**Published:** 2025-09-12

**Authors:** Yasamin Vali, Aquilino Villamonte Chevalier, Henri van Bree, Bart J. G. Broeckx, Ingrid Gielen

**Affiliations:** 1 Diagnostic Imaging, Department of Small Animals and Horses, University of Veterinary Medicine, 1210 Vienna, Austria; 2 Department of Morphology, Imaging, Orthopedics, Rehabilitation and Nutrition, Faculty of Veterinary Medicine, Ghent University, 9820 Merelbeke, Belgium; villamonteaquilino@gmail.com (A.V.C.); henri.vanbree@ugent.be (H.v.B.); ingrid.gielen@ugent.be (I.G.); 3 Department of Veterinary and Biosciences, Ghent University, 9820 Merelbeke, Belgium; bart.broeckx@ugent.be

**Keywords:** computed tomography, elbow joint, elbow dysplasia, medial coronoid disease, radiography

## Abstract

Diagnostic imaging is a crucial non-invasive tool for evaluating canine elbow joints, particularly for screening and treatment planning of medial coronoid disease (MCD). Radiography remains the most accessible and widely used screening modality, but its diagnostic accuracy varies significantly. Sensitivity values reported in previous studies vary depending on factors such as study population, imaging technique, and interpretation criteria. This study compared four measures (sensitivity, specificity, positive and negative predictive value) of radiography with a more advanced imaging method, computed tomography (CT), which provides more detailed images of bones. The results showed that radiography correctly identified 65% of the cases (17/26), while missing some of the affected joints. Furthermore, 7% of the healthy joints (29/398) were misdiagnosed as diseased, leading to a specificity of 93% (369/398). For a disease prevalence of 6% (26/424), the positive and negative predictive value of radiography were 37% (17/46) and 98% (369/378), respectively. Overall, when CT is available, it is the preferred technique for screening. In cases where it is not available, it is important to have an idea of the sensitivity and specificity, but also of the positive and negative predictive value, especially at low prevalences. These results help veterinarians make better decisions when screening for elbow problems in dogs.

## Simple Summary

Elbow diseases are common in dogs and can lead to pain, limping, and a lower quality of life.Medial coronoid disease, is often inherited and affects the elbow joint. Early detection is particularly important for dogs trained for service or work. X-rays are often used to check for signs of this disease because they are quick and widely available, but they may miss the disease or suggest problems that are not actually present. In this study, we compared the accuracy of X-rays with CT, an advanced imaging method that provides clearer bone details. The current study revealed that X-rays correctly identified some of the affected dogs (65%) but missed others, and in some cases, they mistakenly showed signs of disease in healthy elbows (7%). These findings show that CT is a better option when available. When it is not, knowing how often X-rays findings may result in correct diagnosis can help veterinarians decide how to proceed. This study helps improve how we screen dogs for elbow conditions, especially those being trained to assist people or perform important jobs.

## 1. Introduction

Medial coronoid process disease (MCD) is one of the most prevalent causes of forelimb lameness in large and giant breed dogs and constitutes the most frequently diagnosed component of elbow dysplasia [1]. Its multifactorial origin involves cartilage and subchondral bone pathology in the medial coronoid process, often with concurrent joint abnormalities [2]. Affected dogs typically present with pain, reduced range of motion, and weight-bearing lameness, significantly impairing quality of life and training performance [3]. Early and accurate diagnosis is therefore essential for treatment, long-term management, and the selection of disease-free breeding candidates [4]. Screening programs for inherited conditions, such as hip dysplasia, have already improved the health of canine populations [5,6]. Similarly, reliable elbow screening is critical, especially in working and service dogs, where orthopedic soundness is a prerequisite.

Diagnostic imaging is central to elbow evaluation. Radiography remains the most widely used screening tool [7], but its diagnostic accuracy for MCD is highly variable, with sensitivity ranging from 10% to 98% depending on population, technique, and interpretation [8,9,10]. Importantly, diagnostic values derived specifically from screening populations are scarce.

Where radiography is a modality accepted by International Elbow Working Group (IEWG) for screening [11], in recent years, computed tomography (CT) has gained prominence as a more advanced imaging modality for the diagnosis of MCD. It offers unparalleled sensitivity and specificity, with reported values of 100% and 93%, respectively, for the classification and evaluation of MCD lesions [10]. Unlike radiography, CT allows for the detailed visualization of the medial coronoid process and other elbow structures in multiple planes, enabling the identification of subtle pathological changes that might otherwise go undetected [12]. These include fissures, fragmentation, and subchondral sclerosis, as well as complex cartilaginous and osseous changes that define the spectrum of MCD [8]. Given its precision, CT has become an invaluable diagnostic tool for assessing the full extent of disease and for grading purposes [10].

The significance of early and reliable detection of MCD extends beyond clinical signs and management, it also plays a pivotal role in the selection of suitable breeding candidates and working dogs. In particular, dogs selected for specialized service roles, such as guide dogs for the visually impaired or search and rescue dogs in fire departments, undergo rigorous physical and orthopedic evaluations. Elbow dysplasia, and MCD in particular, can compromise their long-term performance and welfare [7]. Therefore, screening programs used in these populations must prioritize not only accessibility and cost-effectiveness, but also diagnostic reliability.

Radiography, while accessible and cost-efficient, is prone to both false negatives and false positives, which can result in the misclassification of affected or healthy animals [10]. False negatives are particularly concerning in screening programs, as they may allow dogs with subclinical disease to enter physically demanding training protocols, potentially leading to injury, poor performance, or early retirement. On the other hand, false positives may result in unnecessary exclusion of suitable candidates from training or breeding pools, thereby reducing the availability of genetically sound individuals. Thus, understanding the diagnostic performance of radiography in real-world screening conditions, where disease prevalence is typically low and imaging is often conducted on asymptomatic animals, is of vital importance.

Previous studies evaluating the diagnostic utility of radiography have often been based on clinical cases or referred populations [10], where disease is more advanced and thus easier to detect. These settings may not reflect the subtlety of findings in early or mild MCD cases commonly encountered in screening populations. Moreover, breed-specific variations in elbow anatomy and joint conformation can further complicate radiographic interpretation, especially in heterogeneous working dog populations. CT, with its high-resolution cross-sectional imaging, mitigates many of these challenges and enables more consistent detection of early and subtle lesions. However, its use in large-scale screening is often limited by cost, accessibility, and radiation exposure considerations [10,12].

Despite the advancements offered by imaging, arthroscopy remains the gold standard for the diagnosis and treatment of MCD [13]. This minimally invasive procedure allows for direct visualization of the affected joint surfaces and the precise identification of cartilage and subchondral bone lesions [14,15]. Arthroscopic interventions, such as subtotal coronoidectomy, utilize specialized instruments like hand-held curettes, motorized shavers, or osteotomes to remove diseased portions of the medial coronoid process [16]. While arthroscopy provides diagnostic and therapeutic capabilities, its invasive nature limits its utility as a routine screening modality. Given the limitations of radiography and the practical constraints of arthroscopy, evaluating CT as a potential screening tool is of growing interest. While CT is well established for diagnosis, its role in population-level screening, particularly in asymptomatic dogs, requires further investigation [10,17].

Considering the widespread availability and use of radiography as the first-line screening tool, the current study was designed to evaluate the diagnostic sensitivity and specificity of radiography for detecting MCD in a population undergoing elbow dysplasia screening, using CT as the reference standard. A secondary objective was to report the positive and negative predictive values of radiography to allow for a more reliable interpretation of diagnostic outcomes, particularly in the context of disease prevalence within the screened population.

## 2. Materials and Methods

This study was a retrospective design, so no institutional animal care and use approvals were requested officially and a written informed consent was obtained from the owners for the participation of their animals potentially in retrospective studies at the time of admission in the hospital.

The database of the Department of Veterinary Medical Imaging and Small Animal Orthopaedics of Ghent University, Ghent, Belgium was searched between January 2011 and March 2016 for the dogs referred for evaluation of the elbow dysplasia and if the dog underwent both radiography and computed tomography. All the cases were assessed and scored by a certifying scrutinizer of IEWG (IG) and 1 European board-certified experienced radiologist (HVB). The studies were anonymized by using the work-station software Osirix (Osirix Medical Imaging Software version 4.1.2 DICOM viewer, Pixmeo, Bernex, Switzerland). The assessment was done in consensus for each patient as paired sets (left- and right elbows). Elbow joints were scored as normal when no alterations in the medial coronoid process (MCP) were observed in either the radiographic or CT assessments. Radiographs were considered positive for MCD if the outline of the MCP was altered, showing changes in shape or radio-density, the presence of a fragment, or increased sclerosis of the trochlear notch. CT images were scored as pathologic if there were changes in the shape, attenuation, fragmentation, or presence of a fissure line in the MCP. Other pathologic findings included trochlear notch sclerosis or an irregular radio-ulnar joint space.

These imaging and anesthesia protocols are consistent with and closely follow those previously reported by the same authors [18]. Based on the protocol of the department of Veterinary Medical Imaging and Small Animal Orthopaedics of Ghent University for radiography of elbow joints, all the dogs were sedated using dexmedetomidine (0.005 mg/kg of body weight, Dexdomitor, Finland). Three standard radiographic views were taken of each elbow joint, a mediolateral extension, a mediolateral flexion and a craniolateral-caudomedial oblique (Cr15°LCdMO) radiographs using 55 kVp and 8 mAs [18].

Radiographs were obtained using a digital radiography system (EKLIN EDR6, Canon Medical Systems, Netherlands). For mediolateral projections, dogs were positioned in lateral recumbency with the upper limb extended caudally and the head retracted to minimize superimposition. The dependent elbow was placed at approximately 120° for the extended view and less than 45° for the flexed view, with the X-ray beam centered on the medial epicondyle. For the craniolateral–caudomedial oblique (Cr15°LCdMO) view, dogs were placed in sternal recumbency with forelimbs extended cranially, and the examined limb pronated 15°. The beam was centered on the joint space just distal to the medial epicondyle of the humerus, and both elbows were radiographed in this position [18].

Immediately following radiography, dogs were anesthetized to allow CT scanning. Induction was achieved with an intravenous bolus of propofol (2 mg/kg body weight; Propovet, Schering-Plough, Comazzo, Italy), followed by endotracheal intubation. Anesthesia was maintained with isoflurane (IsoFlo, Abbott Laboratories, Queensborough, UK) in 100% oxygen. Dogs were positioned in left lateral recumbency with forelimbs extended cranially, and the head was gently retracted laterally to prevent superimposition and artifacts. CT scans were performed using a four-slice scanner (Lightspeed Qx/I, General Electric Medical Systems, Milwaukee, WI) with 120 kVp, 140 mA, and a 25 cm field of view. Contiguous transverse slices of 1.3 mm thickness were obtained from the proximal aspect of the olecranon to 2 cm distal to the elbow joint using a bone algorithm [18].

The statistical analysis was conducted with R (version 3.3.1, “Bug in your hair”). Sensitivity, specificity, positive and negative predictive values were calculated with the “epiR”-package [19,20]. Computed tomography was considered to be the gold standard method.

## 3. Results

This study involved 212 dogs, consisting of 93 females (44%) and 119 males (56%), with a median age of 21 months (range: 5–108 months) and a median body weight of 29 kg (range: 7–59 kg). The study sample represented a wide range of breeds, with the most frequently represented being Golden Retrievers (48%), followed by Labrador Retrievers (27%), German Shepherds (6%), and mixed-breed dogs (5%). A variety of other breeds were also included (Appendix A).

A total of 424 elbow joints were evaluated using both radiographic and CT imaging. The radiographic assessment revealed that 378 (89%) elbow joints were classified as normal, while 46 (11%) were found to be positive for MCD. In contrast, the CT examination showed that 398 (94%) elbow joints were free from abnormalities, and 26 (6%) joints were diagnosed with MCD.

**Figure 1:**
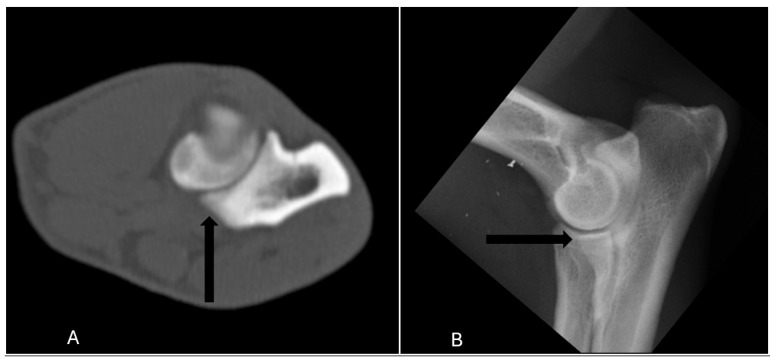
The lateral radiograph and transverse image of the right elbow of a 24 months old German shepherd which was classified as false negative, meaning that medial coronoid process was scored abnormal with a fissure (arrow) only in CT (**A**), and reported normal in radiography (**B**).

**Figure 2:**
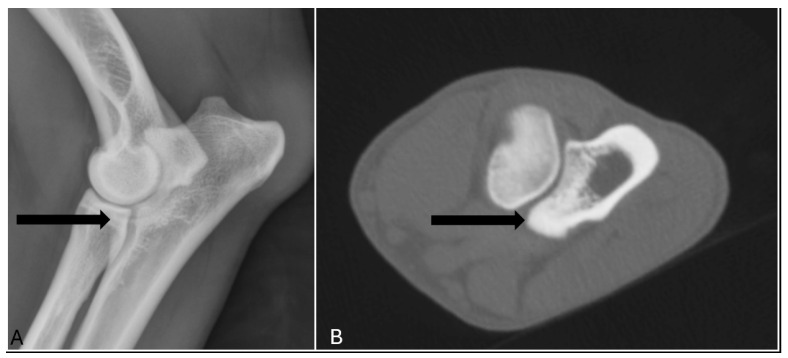
The lateral radiograph and transverse image of the right elbow of a 17 months old labrador retriever which was classified as false positive, meaning that medial coronoid process (arrow) was scored abnormal only in radiography (**A**), and reported normal in CT (**B**).

In terms of diagnostic accuracy, the comparison between radiographic and CT assessments revealed that radiography had a sensitivity of 65% (17/26) and a specificity of 93% (369/398) (Table 1). Specifically, nine elbow joints (9/26) were false negatives, meaning that MCD was detected in these joints only by CT and reported normal in radiography (Figure 1). Conversely, 29 joints (7%) were classified as false positives, indicating that radiography indicated the presence of MCD when in CT no abnormalities were detect. (Figure 2). The positive predictive value was 37% (17/46), while the negative predictive value was 98% (369/378) (Table 1).

Positive likelihood ratio (LR+) was calculated as: LR+ = 1 − Specificity/Sensitivity = 1 − 0.927/0.654 = 8.96. This means that if the radiographic findings are positive (i.e., MCD is indicated), the odds of having MCD increase by approximately 9-fold compared to the pretest odds. Additionally, a negative likelihood ratio (LR−) was calculated as: LR− = Specificity/1 − Sensitivity = 0.927/0.346 = 0.37. This means that if the radiographic findings are negative (i.e., normal joint), the odds of having MCD are reduced to approximately 37% of the pretest odds.

**Figure 3:**
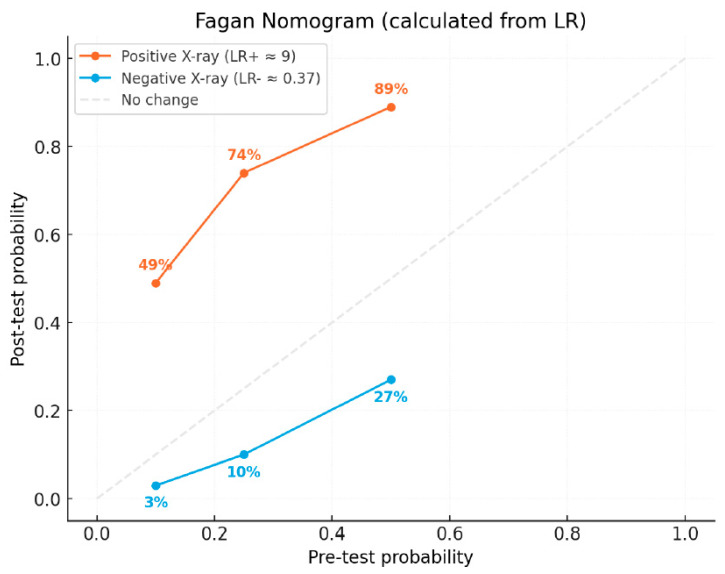
Fagan nomogram illustrating the practical utility of likelihood ratios.

To illustrate the practical utility of likelihood ratios, we used a range of plausible pretest probabilities (10%, 25%, 50%) to calculate post-test probabilities using a Fagan nomogram. A pretest probability of 50% reflects a typical clinical scenario where dogs present with vague signs suggestive of MCD (Figure 3).

In clinical practice, we often encounter patients with vague signs suggestive of elbow dysplasia (MCD), for whom the pretest probability is estimated around 50%. In such cases, radiographic findings can be better interpreted using likelihood ratios. A positive test result (suggesting MCD) increases the post-test probability to approximately 90%, while a negative test result reduces it to around 27%. This demonstrates how radiographic imaging can substantially shift clinical decision-making when interpreted in the context of pretest probability.

## 4. Discussion

The findings of this study demonstrated a relatively low sensitivity (65%) but a high specificity (93%) for radiographic diagnosis MCD, using CT as the gold standard. While the rates of false positives and false negatives were both below 10%, these misclassifications should be carefully considered when interpreting the results.

Our findings, which demonstrate a higher specificity but relatively lower sensitivity of radiography for MCD detection, are consistent with the established advantages of CT imaging. CT’s ability to avoid superimposition allows subtle subchondral bone changes to be recognized more reliably than on radiographs, where summation effects often obscure early lesions. This explains why several cases in our study that were scored as normal radiographically were later confirmed as positive for MCD on CT. Furthermore, while it is generally accepted that cartilaginous lesions cannot be directly visualized by either radiography or CT [21], the close relationship between cartilage and subchondral bone pathology supports our observation that CT was able to detect early bony alterations associated with MCD that radiography frequently missed [22]. Previous reports have emphasized the utility of CT in early screening, especially in predisposed breeds such as Labrador Retrievers, from as early as 14 weeks of age [23,24]. In our study population, which included both typical and atypical breeds, CT similarly proved to be more effective in detecting subtle or incomplete lesions of the medial coronoid process, reinforcing its diagnostic value in heterogeneous screening cohorts. Thus, while CT has been consistently reported as the most accurate technique for identifying MCD lesions [8,13], our results highlight its particular importance in screening contexts where radiography may underestimate disease presence. The sensitivity of radiography for detecting MCD observed in the present study (65%) is consistent with previously published data. For example, Villamonte-Chevalier et al. (2015) reported a similar sensitivity of 64% [10], while Rau et al. (2011) documented a somewhat lower value of 40% [25]. These comparisons suggest that the variability in radiographic sensitivity reported across studies may reflect differences in study populations, disease severity, and imaging interpretation criteria. Our results, which demonstrated a relatively low sensitivity of radiography for detecting MCD, highlight important limitations of the current ED screening scheme recommended by the IEWG. This protocol relies primarily on mediolateral flexed elbow radiographs to evaluate features such as ulnar trochlear notch sclerosis, osteophyte formation, and other primary elbow lesions [11]. While this approach is practical and standardized for large-scale screening, our findings indicate that subtle or early-stage MCD changes are often missed, reducing the overall diagnostic accuracy. This discrepancy suggests that although the IEWG scheme provides a valuable framework for population-level screening, radiography alone may not be sufficient for reliably identifying all affected dogs. Consequently, in cases where radiographic changes are equivocal or clinical suspicion remains high, advanced imaging modalities such as CT should be considered to improve diagnostic confidence. The current study has several limitations that the authors acknowledge, and its findings should be interpreted and applied in clinical settings with caution. This includes not only the imaging methods but also the characteristics of the study population, which can substantially impact both diagnostic accuracy and the generalizability of the results to other settings.

An important aspect to consider when interpreting the results of this study is the specific nature of the screened population. The dogs included in this database were primarily selected for their potential as working dogs, such as those intended for service with fire departments or as guide dogs for the visually impaired. As shown in Appendix A, the majority of the dogs in this study (approximately 75%) were Labrador and Golden Retrievers, which are among the breeds most commonly associated with elbow dysplasia. However, the inclusion of a smaller proportion of less typical working breeds means that some degree of anatomical variability was present, which may have influenced radiographic interpretation. While this variability is unlikely to have had a major effect on the overall findings, it should be acknowledged as a potential contributing factor. Moreover, working dogs often undergo more intensive screening processes at an earlier age and may exhibit less obvious clinical signs despite harboring subclinical joint pathology. This introduces a unique diagnostic challenge, as early or mild forms of MCD may not result in visible radiographic changes but could still be clinically relevant or lead to future lameness. Therefore, the specific demands of this population, especially the need for optimal physical performance and long-term joint health, underscore the importance of diagnostic accuracy and the potential risks of false-negative findings.

A key limitation of radiography is the presence of optical illusions that can affect interpretation [26], leading to a high false-positive rate. Several factors may have contributed to the misclassification of normal joints as MCD-positive in this study. Firstly, the inclusion of a diverse range of breeds may have introduced variations in joint conformation that were misinterpreted as pathological changes.

Additionally, standard radiographic projections may inadequately capture the three-dimensional complexity of elbow joint anatomy, leading to interpretative errors. In breeds with subtle anatomical differences or naturally more prominent medial coronoid processes, these variations could be mistaken for early degenerative or dysplastic changes. This limitation may be exacerbated by the absence of clinical signs or other supporting diagnostic information in screening settings. Furthermore, the 1.3 mm slice thickness in CT assessment, although appropriate for medium to large breeds, may have resulted in some loss of detail in smaller dogs, potentially influencing diagnostic accuracy. However, the marked degenerative changes, if present, would be detected in such cases, the mild changes can remain undetected. It is also possible that the reduced spatial resolution of thicker CT slices in small dogs may limit the identification of incomplete fissures or minor changes in the subchondral bone structure, leading to occasional false-negative interpretations even in CT. However, the evaluation of the diagnostic accuracy of CT was not the primary purpose of the current study, and CT was used as the reference standard due to its superior resolution compared to radiography and its established utility in identifying MCD lesions. Future studies may benefit from the use of high-resolution or cone-beam CT, particularly for evaluating small breeds.

Despite the lower sensitivity of radiography, its high negative predictive value (98%) indicates that it is likely at low disease prevalences, that a diagnosis of “free of MCD” based on radiography, is correct. This makes radiography a useful screening tool in low-prevalence populations, such as those undergoing routine evaluation for breeding or certification purposes. However, the low positive predictive value (37%) underscores the risk of overdiagnosis and the potential for unnecessary exclusion of otherwise healthy dogs from working or breeding programs. In clinical practice, the interpretation of radiographic findings should also be guided by the estimated pretest probability of disease. For example, in patients with vague but suggestive signs of MCD, where the pretest probability can be estimated at around 50%, radiographic outcomes gain greater clinical meaning when assessed using likelihood ratios. Under these circumstances, a positive radiographic result increases the post-test probability of MCD to approximately 90%, whereas a negative result reduces it to about 27%. This highlights that radiographic imaging, although imperfect, can substantially influence clinical decision-making when contextualized with pretest probability estimates. In practical terms, this means that while radiographs can effectively rule out MCD in most healthy dogs, further confirmatory imaging such as CT should be considered when radiographic findings are equivocal.

Comparison with previous studies further emphasizes the variability in radiographic sensitivity based on study population and observer experience. Villamonte-Chevalier et al. (2015) reported a 98% radiographic sensitivity in a population of lame joints, where pathological signs were more pronounced [10], while Carpenter et al. (1993) documented a significantly lower sensitivity of 23.5% [27]. The study by Rau et al. (2011) also demonstrated improved sensitivity (96.7%) when conducted by experienced observers, indicating that diagnostic accuracy is closely linked to radiographic interpretation skills [25]. In our study, the radiographic assessments were performed by two experienced reviewers in a consensus-based approach, which likely contributed to the relatively higher sensitivity compared to some previous reports. However, the use of only two observers may also represent a limitation, as interobserver variability could not be assessed. Nonetheless, other studies employing similar evaluation methods [13,28,29,30,31] have demonstrated consistent and reliable results.

Future investigations could improve upon this limitation by including a larger number of observers and quantifying inter-rater reliability using kappa statistics or intraclass correlation coefficients. Such efforts would help establish clearer thresholds for what constitutes acceptable diagnostic agreement and guide training and standardization efforts in radiographic interpretation.

Another key factor influencing diagnostic accuracy is the study population composition. Unlike some prior investigations that focused solely on clinically affected or referred dogs, the present study included a screening population including a cohort of dogs undergoing routine evaluation for elbow and hip dysplasia. This population structure more closely resembles real-world screening conditions and may explain the lower sensitivity observed, as many dogs had mild or subclinical MCD cases that were challenging to detect radiographically. Notably, our study population comprised a larger sample size (424 elbow joints) and a more diverse range of breeds compared to earlier studies [13,25], which were primarily composed of Labrador Retrievers, Golden Retrievers, and Rottweilers. The inclusion of a wider variety of breeds in our study likely introduced greater anatomical variation, contributing to the observed diagnostic challenges.

These variations in joint morphology may influence the radiographic visibility of MCD-related changes and affect generalizability across different canine populations. Additionally, screening dogs tend to be younger and clinically normal at the time of imaging, which may limit the development of radiographically detectable lesions despite underlying pathology. This age-related limitation also suggests a potential role for longitudinal follow-up studies to better assess the progression of early MCD lesions and the diagnostic window during which radiography becomes sensitive.

Finally, from a clinical and ethical standpoint, it is essential to consider the implications of using an imperfect screening test in populations where the consequences of misclassification can be significant. For instance, a false-positive diagnosis may disqualify an otherwise suitable working or breeding dog, while a false-negative case could result in premature orthopedic failure during physically demanding service tasks. In both scenarios, the welfare of the animal and the goals of the screening program are affected, highlighting the need for an optimized, evidence-based imaging protocol.

## 5. Conclusions

Our results confirm that radiography has limitations as a screening tool for elbow dysplasia, particularly due to its relatively low sensitivity and positive predictive value. When CT is readily available, it is advisable to bypass initial radiographic screening and proceed directly to CT imaging, especially for evaluating elbow joints in the early stages of disease. CT offers more reliable detection of subtle or early pathological changes, which are often missed on radiographs.

In situations where CT is not accessible, a negative radiographic diagnosis should be interpreted with caution, given the possibility of false negatives. This is particularly important when assessing young dogs or those in training for demanding working roles, as undetected early-stage disease may impact future performance or joint health. Awareness of radiography’s diagnostic limitations should guide follow-up planning and monitoring strategies within screening and training programs.

## Figures and Tables

**Table 1:** Number of medial coronoid disease reported positive and negative by computed tomography (CT) and radiography (Rx).

		CT		
		Positive	Negative	
Rx	Positive	17	29	46
	Negative	9	369	378
		26	398	424

## Data Availability

The data and diagnostic images analyzed during the current study are available from the corresponding author upon reasonable request.

## Abbreviations

The following abbreviations are used in this manuscript:

CT	Computed tomography
IEWG	International elbow working group
MCD	Medial coronoid process disease

## Appendix A

**Table A1:** List of dog breeds included in the study.

Breed	Count	Percentage (%)
Golden Retriever	103	48.58
Labrador Retriever	57	26.89
German Shepherd	13	6.13
Mixed breed dogs	10	4.72
Belgian Shepherd	6	2.83
Berger Blanc Suisse	6	2.83
Border Collie	5	2.36
Australian Shepard	2	0.94
Bernese Mountain	2	0.94
Pyrenean Shepard	2	0.94
Bearded Collie	1	0.47
Brittany	1	0.47
Flatcoated Retriever	1	0.47
Goldendoodle	1	0.47
St. Bernard	1	0.47
Standard Poodle	1	0.47
